# Coronaviruses construct an interconnection way with ERAD and autophagy

**DOI:** 10.2217/fmb-2021-0044

**Published:** 2021-09-01

**Authors:** Aref Movaqar, Atieh Yaghoubi, SA Rahim Rezaee, Saeid A Jamehdar, Saman Soleimanpour

**Affiliations:** 1 Antimicrobial Resistance Research Center, Mashhad University of Medical Science, Mashhad, Iran; 2 Department of Microbiology & Virology, Faculty of Medicine, Mashhad University of Medical Sciences, Mashhad, Iran; 3 Inflammation & Inflammatory Diseases Research Center, Faculty of Medicine, Mashhad University of Medical Sciences, Mashhad, Iran

**Keywords:** autophagy, coronaviruses, COVID-19, ERAD, SARS-CoV-2, viral infection

## Abstract

Coronaviruses quickly became a pandemic or epidemic, affecting large numbers of humans, due to their structural features and also because of their impacts on intracellular communications. The knowledge of the intracellular mechanism of virus distribution could help understand the coronavirus’s proper effects on different pathways that lead to the infections. They protect themselves from recognition and damage the infected cell by using an enclosed membrane through hijacking the autophagy and endoplasmic reticulum-associated protein degradation pathways. The present study is a comprehensive review of the coronavirus strategy in upregulating the communication network of autophagy and endoplasmic reticulum-associated protein degradation.

The coronaviruses family has a wide range of strains, which have caused some famous pandemics and epidemics since 2002. As a new member of this family, SARS-CoV-2 has become a major health problem in the world since late 2019 [1]. The coronavirus family has special abilities to replicate inside the infected host cells that make them a skillful family to global epidemics along with the Orthomyxoviridae [2,3]. This family can use some intracellular pathways to increase their proliferation, and following the infection of a cell, all the members of the coronavirus family use an intracellular communications network to replicate themselves. Evidence suggests that the virus can recruit cellular processes to take advantage of its reproduction [4]. Autophagy and endoplasmic reticulum-associated protein degradation (ERAD) are well known as the two most critical pathways for the clearance of misfolded and/or aggregated protein [5]. Furthermore, they have cross-talk with their outer marker membrane through LC3 [6]. Coronavirus has a high ability to use this bilayer membrane to proliferate via preventing binding to lysosomes (Figure 4) [7]. The present study is a comprehensive overview of the communication network between autophagy, ERAD and coronaviruses (Table 1) .

**Table 1.** Autophagy and endoplasmic reticulum-associated protein degradation under rearrangement by Nidovirales, how to recruit these intracellular pathways by Coronavirus subfamilies.

Subfamily	Genera	ERAD	Autophagy	Vesicles	Mechanism of action	Ref.
β-coronavirus	MHV	Increases EDEMosome	Decreases beclin-1, induces autophagosome	DMV, CM, autophagosome EDEMosome	nsp2 and nsp3 make RTC near to ER and induces EDEMosome, DMV, CM and DMS	[57,58]
	SARS-CoV	Increases EDEMosome	Induces mitoPHagy, prevents cell death (apoptosis), induces autophagosome	DMV, CM, autophagosome EDEMosome	nsp2, nsp3 and nsp6 make RTC near ER to dysfunction ERAD process. PLpro-TM and PLP2-TM induces autophagy	[60,67]
	MERS-CoV	Not evidence about EDEMosome	Increases autophagy and LC3-II convertes, decreases Beclin-1	DMV, CM, autophagosome	PLP2-TM and nsp6 induces autophagosome creation, promotes beclin-1 interaction with STING	[71,133]
	SARS-CoV-2	Promote LC3-I conversion into LC3-II, utilized EDEMosome	Increases autophagosome formation, prevents autolysosome	DMV, CM, autophagosome EDEMosome	nsp6 could induces autophagy machinery, nsp3/4 induces DMV construct	[50,62,76]
γ-coronavirus	IBV	Decreased EDEMosome, promote LC3-I conversion into LC3-II, product DMS	Increases autophagosome, prevents autolysosome	DMV, CM, DMS, autophagosome	nsp6 induces autophagy with omegasome intermediate, IRE1 and MAP kinase modulate autophagy, autophagosome diameter shrinks to prevent merging	[50,73]
α-coronavirus	TGEV	Causes the accumulation of misfolded protein, increases ERAD activity	Induces mitoPHagy, prevents cell death (apoptosis), induces autophagosome	DMV, autophagosome	Nucleocapsid could induces mitoPHagy^†^	[74]
	PEDV	Reduces EDEMosome, promote LC3-I conversion into LC3-II	Increased autophagy, induced P53 signaling	DMV, CM, autophagosome	ORF3 inducing conversion of LC3-I to LC3-II and autophagy	[75]
Nidovirales	JEV	Slightly increases EDEMosome in the early steps of infection, replication depend on EDEMosome and LC3-I	Sharply increases autophagosome in the early steps of infection, not required for autophagy and LC3-II	Autophagosome EDEMosome	nsp6 causes strong increases in autophagosome via detected LC3-II	[62]
	EAV	Increases EDEMosome	Induces autophagy	Autophagosome EDEMosome	nsp3, nsp4 and nsp6 causes the production of EDEM-1 turnover, nsp-6 led to rising autophagosome	[58,59]

† Mitophagy: a regeneration and clearance process of mitochondria that preserves quality and function of this organelle.

CM: Convoluted membrane; DMS: Double-membrane spherule; DMV: Double-membrane vesicle; EAV: Equine arteritis virus; ER: Endoplasmic reticulum; ERAD: Endoplasmic reticulum-associated protein degradation; IBV: Infectious bronchitis virus; IRE: Inositol-requiring enzyme 1; JEV: Japanese encephalitis virus; MERS-CoV: Middle East respiratory syndrome coronavirus; nsp: Nonstructural protein; ORF: Open-reading frame; PEDV: Porcine epidemic diarrhea virus; RTC: Replication and transcription complex; TGEV: Transmissible gastroenteritis virus.

## ERAD & autophagy process machinery

### The ERAD process causes the removal of misfolded protein

**Figure 1.**
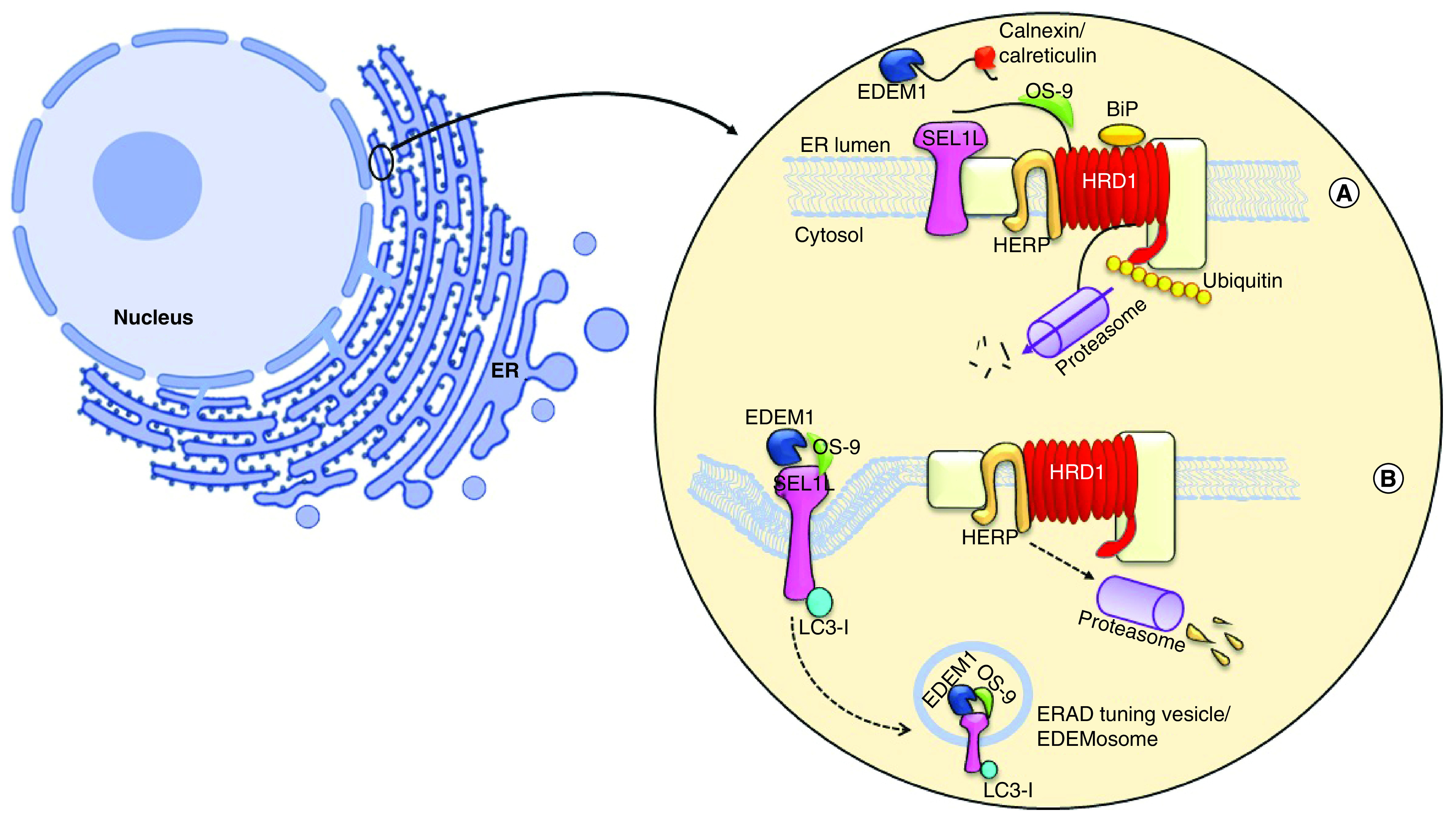
Endoplasmic reticulum-associated protein degradation and endoplasmic reticulum-associated protein degradation-tuning vesicles process. **(A)** The ERAD process contains three major stages: in recognition, calnexin and calreticulin were binding to misfolded protein along with EDEM1, which responsible to remove mannose residue from polypeptide, then its targeting for OS-9 and forwarding whole complexes to the near of lumen; in retrotranslocation stage SEL1L, Hrd1 and HERB create the transportation complexes with misfolded protein. Then, the polypeptide exits from ER membrane and it undergoes ubiquitination by P97 simultaneously. In final stage, the ubiquitinated protein degrade via proteasome. **(B)** A short half-living ERAD regulators or low amount of misfolded protein causes to segregate a transport vesicle from ER, which has coating with LC3-I. The OS-9 and EDEM-1 is budding while LC3-I attaching noncovalently to SEL1-L and construct EDEMosome, the vesicle to be formed deliver to lysosome. ER: Endoplasmic reticulum; ERAD: Endoplasmic reticulum-associated protein degradation.

A conserved process in the mammalian cells that is responsible for the disposal of the misfolded protein in the endoplasmic reticulum (ER) lumen is recognized as the ERAD [8]. This process detects misfolded proteins and extracts them through the ER membrane to cytosolic proteasome. The inability of the ERAD process to demolish the abnormal protein leads to serious illnesses, such as Alzheimer’s disease, Parkinson’s disease, cystic fibrosis, infectious diseases, diabetes and cancer [9]. Overexpression of the nonefficient folding of proteins in the ER lumen accumulates them to be recognized by the ERAD receptors, and then leaves the lumen by retrotranslocation to be degraded by the proteasome [10,11]. Protein transfer, as the base of the ERAD process, can occur from both sides of the transmembrane protein, which is connected to the signal recognition particle (SRP); actually, the nascent protein attached to the SRP could release into the translocation complex of ER, and also SRP could find ER membrane for misfolded proteins [12]. However, some other proteins have independent SPR pathways transferred through the lipid-anchored protein [13–15]. Polypeptide bounds to the chaperone that exists in the ER lumen to change the oligomerization for binding the immunoglobulin protein (Bip), EDEM-1, OS-9, calnexin and calreticulin, altering the conforming structure of Bip to provide a pathway for the protein transfer through retrotranslocation into the cytosol [16,17]. The suppressor/enhancer of Lin12-like (Sel1L) is a partner of reverse transport channel and links to EDEM-1 or OS-9 docking on a recognized polypeptide. Finally, the ERAD substrate is exported from the ER lumen, marked by polyubiquitin chains through P97 hexamer and transported in the center of the proteasome as all the stages are shown in Figure 1 [18].

### ERAD tuning by EDEMosome formation, a vesicle similar to the autophagosome

The ERAD process is tuned by the disposal of its own regulating factors through the formation of vesicular or autophagosome structure to reach the lysosome stage [19]. Some of the folding factors existing in the ERAD process are long-lived proteins, notwithstanding the ERAD regulators, including EDEM-1, SEL1-L and OS-9 that have a short half-life and rapidly demolish from the ER lumen [20,21]. These proteins, participating in the retrotranslocation complex in the ER lumen, make 200–800 nm vesicles and the budding from the ER membrane termed EDEMosome, in other words, the segregation of ERAD regulatory factors are not required for sending the signal to the nucleus occurring with the enhancement or constraint of ERAD activity. A low concentration of misfolded proteins makes the ERAD regulators leaving the ER via vesicles budding (Figure 1) [22,23]. Therefore, EDEM-1 and OS-9 bind to SEL1L and are exported as the segregating vesicles into EDEMosome. The electron microscopy investigation on EDEMosome structure revealed that the ERAD-tuning vesicles are coated with nonlipidation LC3 [24,25]. LC3-I directly binds to SEL1L noncovalently and is located on the outside of this vesicle. This protein is floating in the cytosol environment and is in the form of ubiquitin-like modifier, in other words, LC3-II 27. Finally, the EDEMosome is merged with the lysosome to degrade its contents, as exhibited in Figure 1 [26].

### Autophagy removes the misfolded protein & organelle

**Figure 2.**
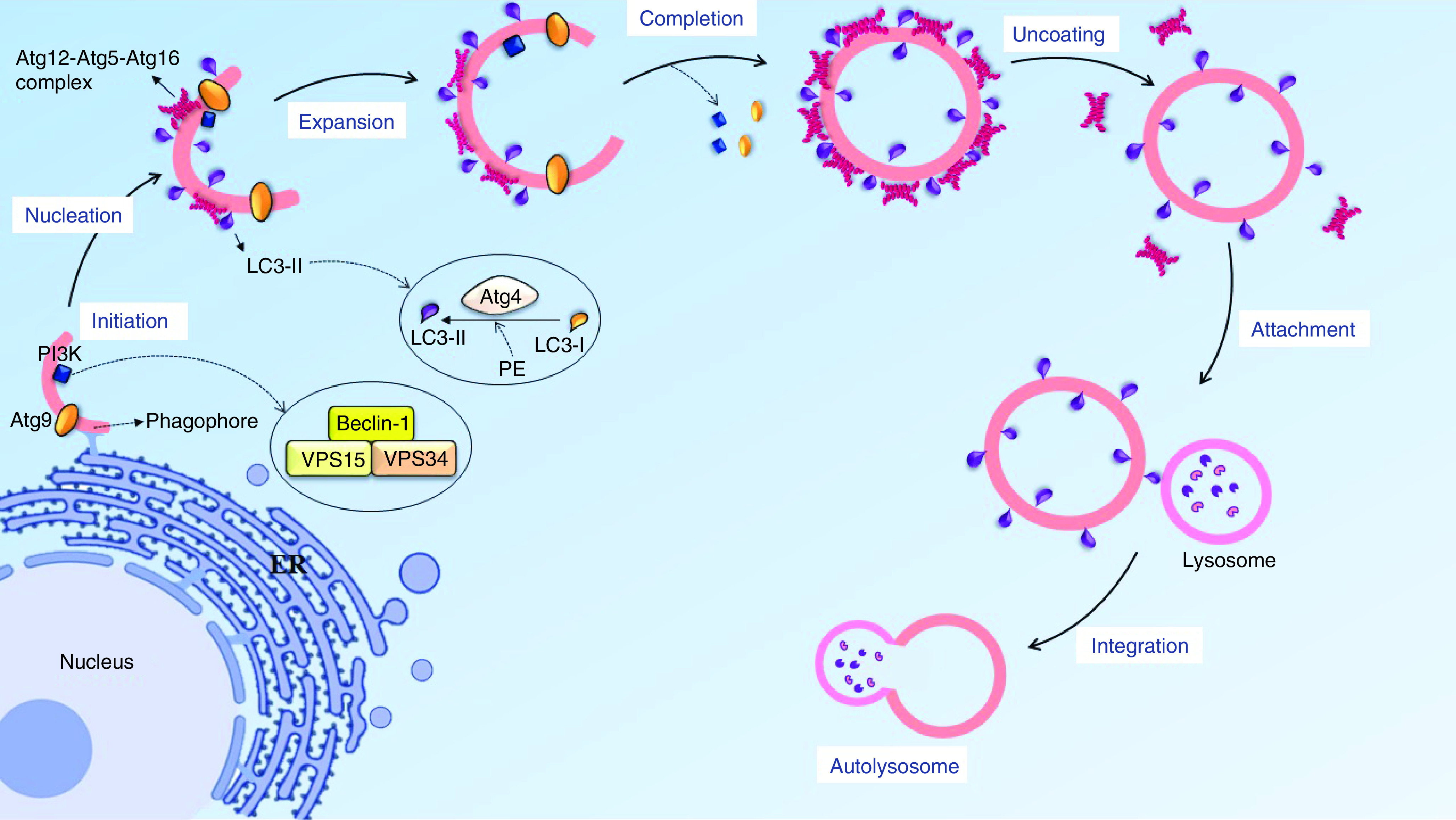
The progression of the autophagy process. Autophagy initiates by sequestration of the lipid membrane from intracellular organelle like endoplasmic reticulum, Beclin-1 and atg9 could knock together a lipid collection machine to extend the lipid bilayer. In nucleation stage, atg12-atg5-atg16 complex is located near the phagophore surface to protected lipid bilayer from integration with other vesicles and also have the E3 conjugating system for LC3-II to progress LC3-I conversion, the lipid bilayer turns into phagosome and then another autophagy factor separates from the vesicle, with the exception of LC3-II. In the final stage, the autophagosome and lysosome could migrate on a microtubule and attach together via the SNARE protein and LC3-II marker.

Autophagy is an intracellular pathway, acting as a regulatory mechanism in the cells to clear unnecessary components or misfolded proteins by creating an enclosed bilayer membrane [27]. This process is initiated by sequestration of the plasma membrane (PM) from intracellular organelles, such as ER, as the primary autophagy structure (PAS) to be constructed by autophagy regulating-9 (Atg9) and phosphatidylinositol 3-kinase complex (PI3K), which exist in the cytoplasm, and both of them are located on a portion of the lipid isolated from the ER [28–30]. Then, the molecular cycle of Atg9 leads to conducting the lipid toward the PAS, and extended to create the primary structure of autophagy termed ‘phagophore’. This structure includes LC3-II, Atg9 and PI3K proteins. Furthermore, the Atg12-Atg5-Atg16 protein complex performs a protection role to keep the phagophore away from being merged with another intracellular bilayer membrane. After the phagophore structure is completely turned into an enclosed bilayer membrane named ‘autophagosome’, the components of the autophagic proteins separate from the autophagosome and the uncoating process will occur. However, LC3-II remains attached to the outer and inner membranes [31–34]. LC3 is known as an autophagic marker due to its binding to the outer membrane of the phagosome (Figure 2). Moreover, one of the most important components of PI3K complex is Beclin-1, which plays an essential role in maintaining the bilayer structure, gathering the autophagy components, integrating with the lysosome and also having a close connection with apoptosis, to be used as a marker for inhibiting or increasing the autophagy [35–38].

### Role of LC3 conversion in ERAD & autophagy activity

LC3-I is a nonlipid form of LC3, which is found in the cytosol, and LC3-II is the modified arrangement of LC3-I that can bind to the autophagosome membrane covalently. These modifications occur in the conjugating system, including glycine adding to the C-terminus region of LC3 and also binding covalently to the amino group of phosphatidylethanolamine (PE) to make LC3-II. These variations lead to the binding of LC3-II directly to the outer region of the autophagosome membrane. The Atg-4 plays a crucial role in the conjugating system of LC3-II in the general process of autophagy [39]. Furthermore, after the formation of autolysosome, LC3-II on the outer membrane will be recycled back into LC3-I by Atg-4-mediated delipidation to redetach from the autophagosome surface to begin another autophagosome formation. Besides, LC3 requires Atg7, Atg3 and Atg5 complex as the E1, E2 and E3 conjugating system to convert LC3-I to LC3-II for the autophagy progression [40]. The autophagy progress is induced in the cell and promotes its activity, causing further conversion of LC3-I to LC3-II and keeping LC3-II intracellular concentration higher than the normal condition, due to increasing the Atg3, Atg7 and Atg5 complex activity. On the contrary, inhibition or constrain of Beclin-1, Atg4 and the other autophagy hotspot genes or the restriction of autophagic activity via bafilomycin or 3MA leads to an escalation of LC3-I accumulation in the cytosol.

## Coronaviruses can rearrange autophagy & ERAD process

### Coronavirus requires an isolated & safe place

After entry and uncoating its membrane, coronavirus releases dsRNA subject and translation of two open-reading frames (ORFs), in other words, *ORF1a* and *ORF1b*. The produced nonstructural proteins (NSP) form the viral replication and transcription complex (RTC) in the preliminary stages [41,42]. There is a harmonic collection of NSP expression along with dsRNA synthesis toward initiating the structural protein expression, viral genomic RNA replication and transcription of subgenomic mRNAs. Mediated dsRNA needs an enclosed platform to cover the incoming genome to avoid the immune system response and also keep itself away from the cellular degradation enzyme [43,44]. This isolated structure is constructed in the ER in different styles, including double-membrane vesicles (DMVs), convoluted membranes (CMs) and open double-membrane spherules (DMSs). According to various reports, coronaviruses are the only family that can produce all three types of vesicles. Previous data showed that infectious bronchitis viruses (IBVs) were the only coronaviruses that could produce the DMS, while other members of the family could produce CM and DMV, separately [45,46]. Also, α-coronaviruses, such as NL63, can develop ER membrane to form complicated clusters, while β-coronaviruses produce all types of organelles to support the RTC complex [47,48]. It seems that the replicative organelles are constructed by coronaviruses in different shapes, but DMV architecture indicates the joint design between their strains. The DMV morphology consists of a bilayer membrane separated from the ER that completely detaches from it and forms an enclosed ring. This morphology is very similar to the EDEMosome and autophagosome appearance [49].

Recent evidence about SARS-CoV-2 revealed that NSP- 3, 4 and 6 have the most important roles for DMV formation and it is predictable that any restrictions in the function of these proteins or blocking their expressions can lead to defective production of the encapsulated bilayer membrane and eventually prevent SARS-CoV-2 propagation [50,51]. In addition to the effective role of NSP-6 in the production of DMV, NSP-6 can increase the Beclin-1 accumulation as well as the precursor structure of omegasome to produce more autophagosome structure. Thus, NSP-6 plays an important role in increasing autophagic activity [52]. The images obtained from SARS-CoV-2-infected tissues by the electron microscopy showed perinuclear DMVs. These data suggest that SARS-CoV-2 needs a security guard, similar to the rest of its family, to stay away from the responsive effects of the immune system and cellular enzymes. Thus, the virus tends to create an enclosed space with a bilayer membrane taken from the ER. There is limited evidence about the morphology and type of vesicles that SARS-CoV-2 obtains from the ER, but regarding the discussions about the coronavirus family, it can be predicted that SARS-CoV-2 can construct DMV and CM, as the other members of β-coronaviruses [53].

A cell infected with coronavirus under the influence of viral proteins makes the ER membrane expansion and occupies a large volume of the cell. Furthermore, increased accumulation of viral-expressed proteins along with cellular misfolded proteins within the ER lumen and cytosol leads to increased activity of cellular homeostasis pathways such as ERAD, autophagy and another cytoprotective signaling pathways like unfolded protein response (UPR) [54]. Cells infected with coronaviruses-enhancing ER capacity to remove misfolded proteins accumulation or aggregation, reducing ER load and protein flux and also activate cell death program. The ER capacity were enhanced by activating the ERAD pathway using EDEM-1 and PDI chaperones that led to ER stress relief and eliminates intracellular protein accumulation. In addition, the cell also tends to phosphorylate more eLF-2 to reduce the protein flux in addition to stopping newly protein synthesis in the cell. Eventually, this ER stress by activating Bcl-2, CHOP and JNK leading to autophagy and apoptosis. Coronaviruses creating a bilayer membrane derived from ER to staying out of the reach of intracellular enzymes, in addition, can act as a regulator of cellular stress by recruits ERAD and chaperon production to prevent the cell from going to ‘complete autophagy’ or ‘enzymatic digestion’ in the area where the virus multiplies [55,56].

### Coronaviruses utilize EDEMosome as an enclosed safe scaffold for their replication

Some of the positive RNA viruses use the ERAD-tuning process for their propagation. EDEMosome provides an appropriate enclosed structure for virus translation, transcription or replication. The virus can also get away from detection by the immune system. SARS-CoV and the mouse hepatitis virus (MHV) increase the ERAD-tuning vesicles. It has been shown that the amount of EDEM-1 accumulated in the cells infected by MHV and SARS-CoV was significantly increased, and also MHV replication reduced in LC3-I knockdown cells, which develop the defective type of MHV without NSP-2 and NSP-3 portion of the virus particle [57]. The knockdown of the siRNA-mediated SEL1L receptor cells can reduce the acceleration of the MHV propagation. Also, the equine arteritis virus (EAV) has a similar manner in utilizing the ERAD-tuning vesicles by increasing the EDEM-1 turn over from the ER lumen and rising the EDEMosome vesicle in the infected cells. The electron tomography demonstrates the coexpression of NSP-3 and NSP-4 in SARS-CoV and the MERS-CoV to induce DMV vesicles. Furthermore, numerous DMV and CM are found near the nucleus in Huh7 cells infected by MERS-CoV [58,59].

The consequences of coronavirus infection in the ERAD pathway indicate the formation of an encapsulated bilayer membrane covered by LC3-I. Besides, the loss of LC3 will prevent the formation of DMV. Thus, preserving LC3 is essential for the ongoing virus replication [58,60]. The role of LC3-I in the MHV infection needs more attention since *Atg5* and *Atg7* were deleted from the MHV-infected cells, showing no effective role for the considered replication. On the other hand, MHV can keep its ability for propagation even in the block autophagy conditions. LC3-I has a critical role in the replication of MHV, and the removal of the autophagy hotspot gene does not prevent the virus reproduction. The same is true of the Japanese encephalitis virus (JEV), and the suppression of JEV replication has been observed via the SEL1L and EDEM1 expression shutdown. Indeed, JEV cannot resume its proliferation without releasing EDEMosome. Also, LC3-I depletion causes the reduction of the viral load of JEV in the infected cells [61].

*In vitro* investigation about LC3-I and LC3-II expression levels reveals that in the earlier steps of infection, the expression of LC3-II reaches its highest level compared with the beginning of the infection, and LC-I shows a slight increase. Hence, the cell’s natural response to the onset of infection, which leads to an increase in the autophagy activity relative to the infection stress (to be discussed later in Section 3.3, as the coronaviruses increase the rate of autophagy to use autophagosomes more than the state of cell infection) along with increasing LC3-I production in constructing DMV and CM structure to help the virus propagation indicate the essential need of the virus to form the encapsulated vesicles [62]. The assessment of some Nidoviral member actions shows that JEV and EAV utilize the regulatory process of the ERAD and use its internal space for proliferation, which is suggested to be due to a common conserved region in 3CLpro and TM. The coronavirus family may exhibit a different way of acting in the γ-coronavirus subfamily and the IBV genus, causing the reduction of the EDEMosome production and preferring to promote the autophagy activity for its proliferation. Moreover, the α-coronavirus subfamily and PEDV genera adopt a similar mechanism of IBV to induce autophagy instead of increasing the ERAD-tuning vesicles by increasing the conservation of LC3-I to LC-II. The porcine epidemic diarrhea virus (PEDV) uses *ORF3* to increase the LC3-II production, and the IBV does similar action by expressing *NSP-6*. Despite the different behaviors of α/γ-coronaviruses, the cases investigated under the β subfamily reveal the induction of EDEMosome and DMV formation by *NSP-2* and *NSP-3* expressions. SARS-CoV and MHV develop RTC near the ER to improve the ERAD-tuning vesicles budding and dysfunction of the ERAD process. The high similarity of the NSP-2, NSP-3 and NSP-4 of SARS-CoV-2 with SARS-CoV can give us a clue as to how SARS-CoV-2 affects the ERAD-tuning process. SARS-CoV-2 also needs to have an enclosed region to replicate its intermediate RNA to direct it toward using the ERAD pathway [63].

Some viruses manipulate the ERAD pathway to advance their infection. HCV reduces the amount of its E2 glycoprotein via activating the ERAD pathway, which avoids to display them on the cell surface for recognition by the adaptive immune system [64]. Also, inhibition of EDEM-1 leads to interfere E2 ubiquitination of HCV and constrain its replicate, a similar event was found for HBV surface glycoprotein that also uses ERAD to replicate itself and utilized EDEM-1 for folding its glycoprotein. The same is true of HIV, the Vpu protein of the virus can obtain E3 as a target for recruit ERAD and lead to its ubiquitination, also, ERAD activity protects the HIV from being targeted by natural killer cell (NK) [65]. However, another virus tends to escape from ERAD or reduce its activity, upon entering the ER via endocytosis, the polyomaviruses attach to the PDI chaperone and exit the ER through the Derlin-1,2 using a retrotranslocation mechanism next bind to the nuclear pore and following uncoating occurs, then enters DNA into nucleous. However, the coronavirus family has been little identified as being able to use the ERAD regulatory mechanism for replication. Coronaviruses can use the ER-released bilayer membrane to amplify their intermediate RNA. Also, as mentioned in Section 3.1, the spatial forms resulting from cell infection with coronavirus include DMV vesicle, which is coated with LC3-I and similar to EDEMosome surface marker, coronaviruses utilized both vesicles to multiply [65,66].

### Coronaviruses & autophagy

Preliminary research about MHV coronavirus has shown that the infected cells have more autophagy activity than the normal ones. Nevertheless, based on the last section, MHV and SARS-CoV keep their ability to replicate in the mutant (Δ*Atg-5* and Δ*Atg-7*) cells [67,68]. Since two genes remove from the cell involved in the LC3-II conjugating system (see the previous section), the degradation and defect of autophagy function have no significant effects on the coronavirus replications [69,70]. MERS-CoV infection prohibits the completion of the autophagy process by preventing the formation of autolysosome through NSP-6 expression since it has a key role in increasing the autophagosome formation, whereas MERS-CoV reduces Beclin-1 intracellular concentration level. Evidence suggests that the overexpression of membrane-associated papain-like protease PLP2 (PLP2-TM) in SARS-CoV and MERS-CoV causes inhibition of the autophagosome integration with the lysosomes while increasing the amount of LC3-II in the cell [71,72]. Other studies demonstrated that the induction of Beclin-1 formation by NSP-6 expression in the cells affected by the IBV was along with preventing the autolysosome formation. According to the above, MERS-CoV can increase the rate of autophagy in the cells while decreasing the concentration of Beclin-1. It differs for IBV, which increases autophagy by increasing the Beclin-1expression. Thus, we observe two different functions of two different strains of the coronavirus family encountered by Beclin-1. Furthermore, detection of LC3-I and LC3-II in the mouse embryo fibroblast infected by the IBV shows that expression of NSP-6 can induce releasing autophagosome rather than EDEMosome [50,73]. IBV causes the autophagosome diameter to shrink to prevent merging with the lysosome. Similarly, the transmissible gastroenteritis virus (TGEV) and SARS-CoV can induce autophagy through mitochondria and keep themselves away from the oxidative stress response, which leads to cell death. Negative regulation of autophagy is time dependent for TGEV, and the autophagosome increase can be found by LC3-II detection in the early steps of TGEV replication. However, the same increase in autophagy can limit the acceleration of virus replication in the late stages [74].

As another member of the α-coronaviruses, the PEDV has expression of ORF3, inducing the autophagy through the conversion of LC3-I to LC3-II and reducing the EDEMosome construction as in the IBVs. The autophagy device for screening LC3-II production of the infected cells is the transmission electron microscopy [75]. The α-coronavirus members have two different acts encounter to reactive oxygen species (ROS) influences, PEDV can induce cell death through mitochondrial-activating factors by using the *ROS/P53* pathway, furthermore, the S1 protein can also act as an inducer of apoptosis, whereas TGEV constrains the inhibitory effect of the ROS pathway by activating autophagy in the early stages of infection. Coronaviruses tend to increase their autophagic activities in the cells before merging with lysosomes. The results of several studies show that the lack of autophagy genes does not prevent the coronavirus from replicating. However, coronavirus can increase the autophagosome formation and stop it at the preceding stage of maturation.

*In vitro* studies show the inhibition of autophagy activity in the cells infected with SARS-CoV-2 by mTOR functional promotion. Another study evaluated the cytopathic effect of autophagy modulators in the Vero-E6 cells, which had a different target in the autophagy pathway. In this regard, the researchers concluded that the cells under treatment with the negative modulators of autophagy had less cytopathic effect than the others [76]. With all the evidences concerning the increased activity of the autophagy device due to the coronavirus family, it should be noted that a definite claim about the relationship between SARS-CoV-2 and autophagy requires more accurate studies about the virus itself, and investigations should be expanded to the level of sampling from the COVID-19 patients.

**Figure 3.**
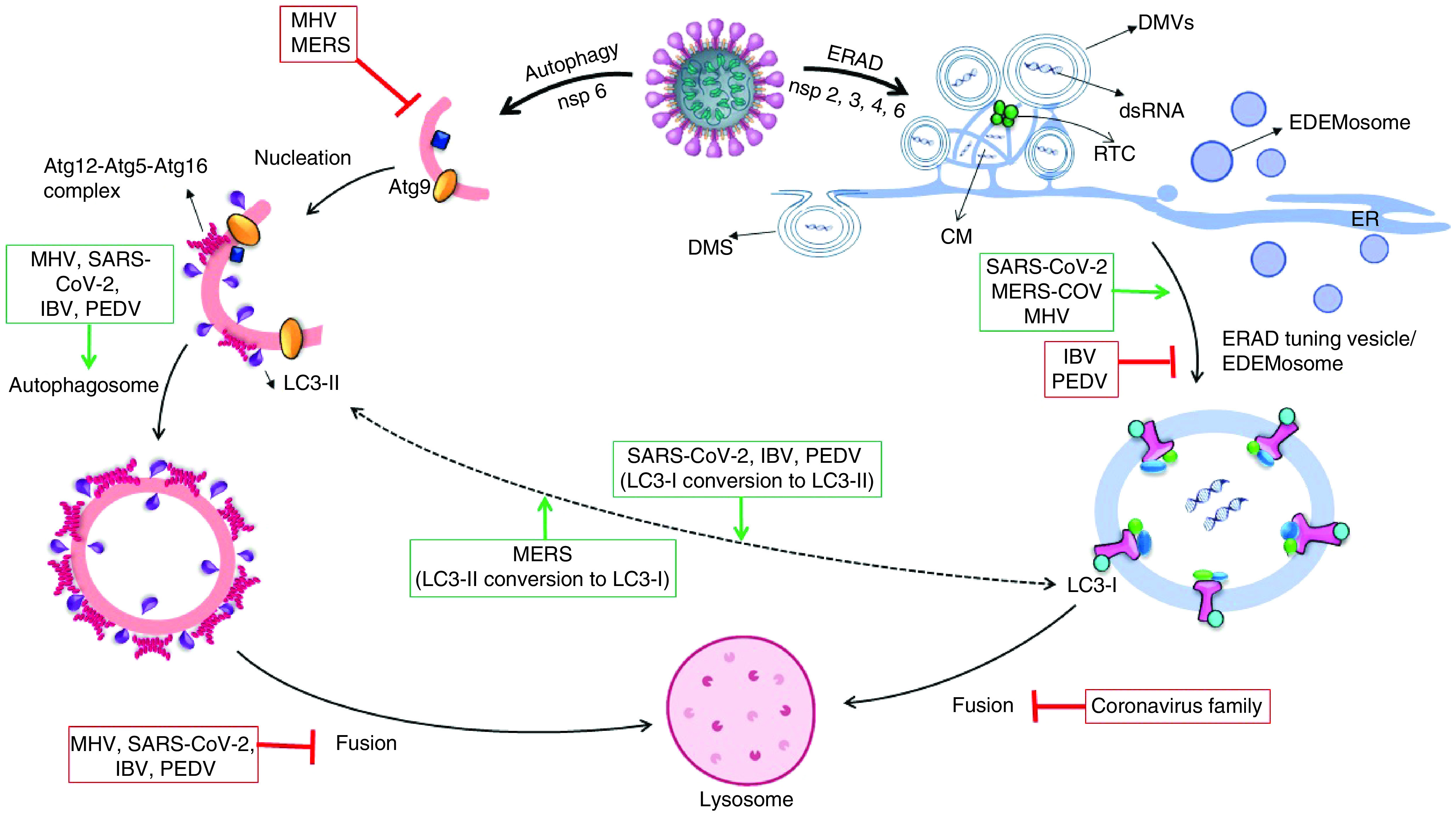
In replication of coronaviruses: after genome uncoating and produce mediate dsRNA, it needs an enclosed region for transcription as well as completion of its proteins. Coronaviruses can construct three types of enclosed chambers with the recruits of ER membranes by the way of RTC making near of ER. DMV is a circular bilayer membrane made by most members of the coronavirus family, including SARS-CoV-2. CM is an irregularly enclosed area that forms a grid and it is formation by most member of coronaviruses. DMS is an omega-shaped membrane caused by the protrusion of a bilayer membrane that only IBV can make. LC3-I:LC3-II concentration ratio is linkage between autophagosome and EDEMosome fabrication level in cells, LC3-I conversion to LC3-II to get ability to putting into lipid membrane, also LC3-II delipidation to LC3-I via atg4 for another autophagosome creation. MERS-CoV is the only member of the coronavirus family that tends to produce more LC3-I. In addition to limiting EDEMosome production, IBV increases autophagosome production and LC3-II construction. SARS-CoV-2 induces ERAD and autophagy by its nonstructural proteins: nsp3, nsp4 with nsp6 cause to promote ERAD-tuning vesicle production and nsp6 lonely could induce autophagy activity. SARS-CoV-2 makes DMV and CM by the way of extending ER membrane or EDEMosome hijacking to translate dsRNA-mediated. Furthermore, SARS-CoV-2 increases autophagosome formation and prevents its fusion with lysosome. CM: Convoluted membrane; DMS: Double-membrane spherule; DMV: Double-membrane vesicle; ER: Endoplasmic reticulum; ERAD: Endoplasmic reticulum-associated protein degradation; IBV: Infectious bronchitis virus; MERS: Middle East respiratory syndrome; nsp: Nonstructural protein; PEDV: Porcine epidemic diarrhea virus; RTC: Replication and transcription complex; SARS-CoV: Severe acute respiratory syndrome coronavirus.

**Figure 4.**
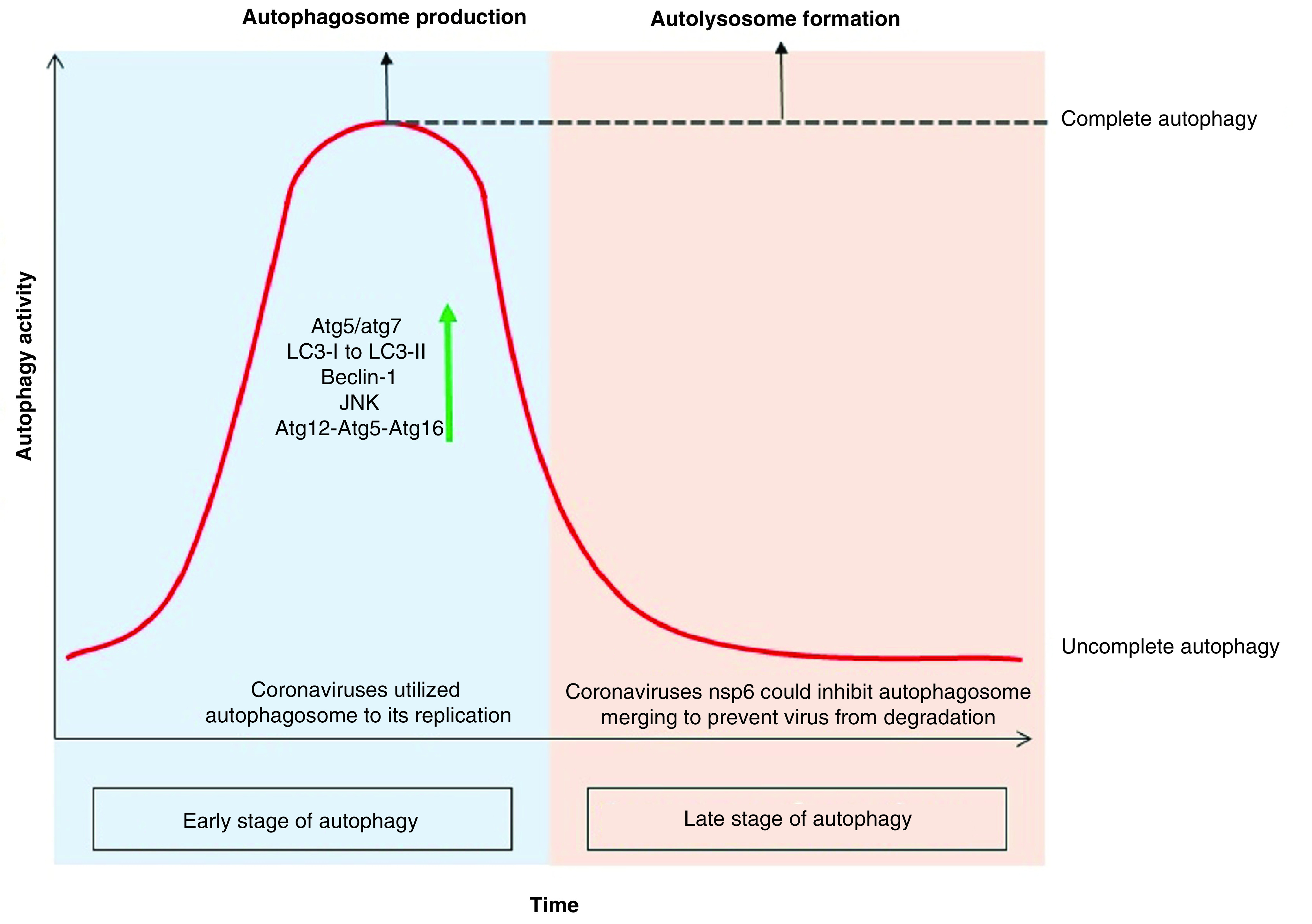
Autophagy activity in time course of coronavirus infection. Since the cell infection with the coronavirus, the autophagy activity has been elevating sharply, including an increase in the production of PI3K components like atg12-atg5-atg16 and beclin-1. It also induces the JNK pathway leading to autophagy, which can be induced by promulgate the xeroderma pigmentosum type B splicing of the inositol-requiring enzyme 1 pathway or by a direct effect on the MAPK. According to the studies, deletion of *atg5* and *atg7* genes reduces coronavirus replication. Coronavirus-infected cells experience increased LC3-I modification due to the addition of glycine to the C-terminal as well as the covalently binding of phosphatidylethanolamine to its amino terminus. This modification results in the production of LC3-II that bind to the outer membrane of the autophagosome and increase the autophagosome marker inner cell. In the late stage of autophagy, coronavirus with the nsp6 infectious protein could constrain autolysosome constructs and turns the cell toward ‘incomplete autophagy’ to keep the virus away from the lysis enzyme of autolysosome.

Based on investigations, coronavirus-infected cells in the early stage of infection experience an increasing in autophagic activity, which can be due to both the cell’s reaction to the pathogen and the fact that the coronaviruses themselves increase the autophagic activity by nsp6. This increase in autophagic activity is limited to the production of more autophagosomes, which in some members, such as MHV, does so without increasing beclin-1 levels [77]. However, in most members of this family, more autophagosome production is associated with increasing levels of Beclin-1, atg12-atg5-atg16 and JNK [78]. But in the next stage of coronavirus infection, they prevent the integration of autophagosomes and lysosomes, which is done by disrupting the receptor at the junction of the two vesicles or by shrinking the size of the autophagosome to constrain the vesicles from joining. In other words, infection of cells with the coronavirus family leads to incomplete autophagy, which is accompanied by a significant increase in autophagosome levels as well as the prevention of cell death while uninfected cells that being under tension tend to increment the complete autophagy process, indeed, an increase in autophagosome is accompanied by an increase in autolysosome production as all shown in Figures 3 & 4 [79].

## Inhibiting & activating of autophagy/ERAD could impact on coronavirus replication

### Autophagy/ERAD inhibitor

Chloroquine (CQ) and its less toxic derivative hydroxychloroquine (HCQ) have received attention for the treatment of SARS-CoV-2 infection. CQ and HCQ are endosome/lysosome regulators, which are essentially required in autophagy, especially in the maturation stage [80]. HCQ is more soluble and less toxic than CQ, and both of them are consumed in phosphate and sulfate forms with a long half-life that is estimated to be between 40 and 60 days [81]. CQ function in autophagy is by interfering with the autolysosome formation through increasing the endosomal/lysosomal pH. The unprotonated CQ to be used is diffused easily across the membrane because of its diprotic weak base, following mono- or diprotonated CQ in the lysosome, leading to the loss of the capacity of diffusion. Likewise, CQ can block autophagy through inhibiting the lysosomal proteasome and preventing the autophagosome maturation. Therefore, CQ is used in combination with chemotherapeutic drugs for cancer treatment [82,83]. The chemical properties of this drug in the elimination of lysosomes and endosomes also lead to inhibition of SARS-CoV-2 entering, uncoating and exocytosis from the cells. Nevertheless, some of the important systematic review and cohort studies have not indicated any evidence that CQ and HCQ are effective in improving the recovery of COVID-19 patients, lessen their symptoms and useful as prophylaxis [84–87]. Based on the related studies, CQ and HCQ seem to be in conjunction with COVID-19 to prevent the completion of the autophagy process, which may support the hypothesis of an increase in the initial titer of SARS-CoV-2 virus in the patients receiving HCQ. This hypothesis is compatible with the results of two studies about SARS-CoV and HIV patients confirming that the patients, who received CQ, had a high initial viral load and also a delay in the initial response of the immune system [88,89].

Lopinavir and ritonavir (protease inhibitors, PIs, with the commercial name of Kaletra) induce the ER stress and oxidative pathway, which impair autophagy activity in melanoma and adipocyte cells, mTOR activity and protein expression enhanced in the skeletal muscle cells under PIs treatment, but the results obtain *in vivo* demonstrate the reduction of mTOR activity in mice [90–92]. PIs *in vitro* research have shown the increase of protein degradation machinery activity up to 150% in comparison to the nontreatment group [93]. In addition, this combination induces ER stress, which is associated with the proteasome degradation of ubiquitin protein [94]. On the contrary, the consequences of ROS triggered by PIs impair mitochondrial activity and ubiquitin-proteasome system dysfunction [95]. Since PI treatment exhibits increasing of ubiquitin proteins significantly along with ERAD dysregulation, this may lead to an accumulation of protein aggregates in the cells [96–98]. There is no evidence about EDEMosome promotion or reduction in the cells treated with PIs. However, it is expected that the consequences of the higher amount of ubiquitin protein, which involve EDEM-1 and other ERAD regulator factor in the ER lumen, causes the reduction of EDEMosome generation. Contradictory reports have been published about the effectiveness of lopinavir/ritonavir (kaletra), in some of which, the effectiveness of inpatient mortality has been appropriately evaluated, and in others, it seems to be significantly useful [99,100]. Two meta-analyses of the administration of kaletra to the patients with COVID-19 report no significant difference in the duration of hospitalization or mortality rates compared with the patients who did not receive the drug [101,102].

### Autophagy/ERAD activator

Dexamethasone (DXM) is a type of corticosteroid, which promotes autophagy activity through the mTOR pathway. Two *in vitro* observations of DXM showed the cells that were incubated with various concentrations of DXM had higher autophagy proportions [103,104]. In comparing the incidence rate of autophagic activity in CD4^+^ T-cell between the control and DXM undertreatment groups, it was concluded that DXM could induce autophagy in the CD4^+^ T lymphocytes of asthma patients [105]. Previously, the correlation between DXM and autophagy was found in muscle atrophy, leukemia and lymphocyte, and osteoblast had been discovered [106–109]. In all of these cases, DXM increases the rate of autophagy through increasing Beclin-1 accumulation or conversion of LC3-I to LC3-II. *In vivo* investigation about the direct relation between DXM and ER stress demonstrates that DXM can inhibit ER stress by the NF-κB-mediated anti-inflammatory action in mice. Moreover, mice treated with DXM had lower misfolded protein accumulation, though glucocorticoids lead to the enhancement of the correct folding protein in the cells, upregulating EDEM-1 and other ERAD regulators for secretion with EDEMosome [110]. DXM modulates lung damage by inflammation and reduces the disease progression of respiratory failure and death. According to the latest research, the patients with DXM treatment have 8–26% lower mortality compared with noncorticosteroid treatment [111,112].

Type I interferon can involve JAK-STAT, PI3K, AKT and mTOR pathways to induce autophagy and antigen presence [113–115]. Accumulation of acidic lysosomes as a result of the beclin-1 pathway, which is possible through autophagy, can be denominated the use of IFN-α2b in the treatment of cancer cells [116]. Furthermore, inhibition of the autophagy pathway in the cells will reduce the IFN-α2b and CXCL10 expression, and also, autophagy was found to be needed for IFN-α production in the dendritic cells [117,118]. Therefore, there is a significant association between autophagy activity and IFN-α production. Due to that respect, autophagy delivers the intracellular pathogens for lysosomal degradation. Therefore, CD4^+^ T cells are considered as a consequence of lysosomal contents loaded onto MHC class II molecules. This process induces self-tolerance of the CD4^+^ T-cell repertoire and led to T-cell responses against pathogens [119]. *In vitro* investigation on hepatocellular carcinoma and glioblastoma cells shows that IFN-β and IFN-γ promote autophagosome formation and conversion of LC3-I to LC3-II [120,121]. The increased ERAD modulators, such as EDEM1, correlate with the proinflammatory high-level expression, and high concentration of IFN-γ has significant interconnection with an upward level of EDEM-1 and SEL1L [122]. However, another study showed negative regulatory of IFN-γ on mRNA expression of Bip, sec61 and no effective role in calreticulin [123]. The delay in IFN-α response in SARS-CoV infection is associated with lung lesions and fatal outcome; preliminary *in vitro* experiments display that SARS-CoV-2 has more sensitivity than SARS-CoV to the IFN-α response and because of IFN-dependent induction response to SARS-CoV-2, type-I IFN is a therapeutic benefit in COVID-19 treatment [124–126]. A meta-analysis study assessment shows that COVID-19 patients under treatment with IFN-α/β have significant viral clearance, with the reduction of the duration of virus detection in the upper respiratory system and elevate the blood inflammatory factors [127]. Furthermore, reduction of IFN-γ circulation may cause lung fibrosis, observed from CT scan imaging, RT-PCR and the blood sample results of the patients [128].

### Dual role on autophagy/ERAD

Bortezomib is an IL-6 receptor blocker used to treat patients with multiple myeloma. The effect of this drug on intracellular signaling makes intracellular calcium ions releases upward, which helps to induce ER stress and autophagy. Bortezomib is also known as a proteasome inhibitor that reduces ERAD activity and causes elevate the accumulation of intracellular proteins [129]. Protein accumulation leads to UPR activation as well as GRP78 and CHOP chaperones. It also induces autophagy by rising JNK activity directly or by increasing XBP splicing in the IRE1 route. In addition, bortezomib constrains JAK-STAT activity by blocking the IL-6 receptor (a function opposite to IFN-α,β activity) and reduces inflammation, autophagy and angiogenesis in cancer cells [130]. This dual function of bortezomib makes it difficult to decide how to play a role in coronavirus replication. The effect of this drug as an anti-inflammatory is used in the patients with COVID-19 who have entered the inflammatory phase of the disease and does not seem to play an effective role in inhibiting the replication of the coronavirus by altering autophagic or ERAD activity [131].

## Conclusion

Coronavirus tends to form a protective shield to keep itself away from recognition and degradation. This virus develops an enclosed space taken from the autophagosome, EDEMosome or another form of ER membrane budding (DMV, CM and DMS). The bilayer membrane morphology of β-coronaviruses seems to be similar in all the members, even SARS-CoV-2 [61]. Based on the studies, all members of the corona family create the DMV structure with the LC surface coating, but some of them like the IBV could create the CM structure. In general, ERAD pathway activity is examined separately from ERAD regulation. Cell infection of the coronavirus family leads to accumulation of viral infectious proteins in the ER lumen, which induce further activity of cell homeostasis pathways, ERAD is used to remove these infectious proteins from the ER lumen and deliver them to the proteasome. The evaluation of some Nidoviral members showed increases in both EDEMosome and autophagosome marker inner cells (LC3-I and LC3-II) during the infection [132]. However, except β-coronaviruses, another coronavirus subfamily revealed a different strategy when encountered with the ERAD process: the IBV, TGEV and PEDV tend to reduce EDEMosome and promote LC3-II modification as opposed to SARS-CoV, MERS-CoV and MHV [57,58,60,74,75,133]. This difference in the effect on ERAD regulatory activity leading to EDEMosome production appears to be due to the different expression of infectious proteins in the β-coronavirus family. *In vitro* findings indicate an increase in EDEMosome production as a result of infection with the β-coronavirus family that it can be expected that this family prevents the accumulation of their infectious proteins in the ER lumen and increases ERAD activity by helping with the ERAD pathway and the production of cell chaperones like GRP78 or PDI [134].

The strategies of coronaviruses to confront the autophagy process are somewhat the same, and they all enhance autophagy before autophagosome maturation [82,103,104,111,112,135]. Some of the coronaviruses such as SARS-CoV-1 and MERS-CoV could induce the autophagy process by PLpro-TM. MERS-CoV inhibits Beclin-1 activity by the way of STING repressor, and in general, MERS-CoV increases autophagosome without Beclin-1 [111,112]. In another word, ‘uncompleted autophagy’ without the last stage of autophagosome integration is beneficial for the coronavirus family and simultaneously coronaviruses need to have inducible autophagy activity to have more accessibility to an enclosed capsule for replication, indeed, they could be hijacking autophagy process after constructs autophagosome to avoid degradation itself by lysosomal enzyme [136].

Although cellular studies on the cross-talk between autophagy and SARS-CoV-2 indicate the limiting role of autophagy for the propagation of this novel virus, the virus can use autophagosome for its reproduction, and it seems this novel virus has a similar reaction to autophagy as the other β-coronaviruses [106–109]. The increasing activity of autophagy by the coronavirus family and definite assertion about the relationship between SARS-CoV-2 and autophagy need more precise and expanded investigations considering the patients in this regard. The remarkable aspect about the communication network between autophagy, ERAD and coronaviruses is that the autophagy and ERAD process is inherently a limiting factor, autophagy could engulf viral component to degrade via lysosome and ERAD causes the infectious protein to degrade by sending it to the proteasome, but coronavirus causes autophagosome recruitment by changing its function in the last step, creating ‘incomplete autophagy’, this process overactivity due to the drug induction may prevent the virus from controlling this intracellular process like DXM and IFN-α,β, all of which has been shown in Table 2 [137]. However, the β-coronavirus subfamily behaves differently from other members in dealing with ERAD and prevents further activity of the ERAD process by reducing the amount of accumulated proteins. Given that there is no further information on how coronavirus infection affects cell chaperone proliferation, the β-coronavirus subfamily appears to help increase cell chaperone production and increase UPR activity. This is probably due to the increased production of DMV and EDEMosome for greater access to ER-derived bilayer structures for self-propagation [138]. While in the case of ERAD tuning, it does not constitute a limiting process in itself for the pathogens, actually it could be providing the opportunity for the virus to multiply further.

**Table 2.** Alteration of autophagy and endoplasmic reticulum-associated protein degradation function under the influence of drugs used in COVID-19 treatment.

Drugs	Autophagy	ERAD	Mechanism of action	Ref.
Chloroquine/hydroxychloroquine	Block the endosome formation	NF	Interfering with autolysosome formation through increasing the endosomal/lysosomal pH	[82,83]
Dexamethasone	Induce autophagy	Inhibit ER stress, decrease ERAD function, reduce misfolded protein	Beclin-1 accumulation, promote LC3-I conversion to LC3-II	[103–105]
Lopinavir and ritonavir (Kaletra)	Impaired autophagy activity	Lead to ERAD dysfunction, accumulation of misfolded protein aggregation	Induced ER stress, trigger ROS pathway	[95,96,139]
IFN-α2b/β	Increase autophagy and antigen presenting	NF	Induce JAK/STAT, induce PI3K/AKT, reduce mTOR pathway	[113–115]
IFN-γ	Increase autophagy activity	Cause to increase SEL1L and EDEM-1	Promote autophagosome formation, increase LC3-I conversion to LC3-II	[120,122]
Bortezomib	Dual effect	Decrease ERAD	Inhibit proteasome and cause to accumulate intracellular misfolded protein/increase JNK, IRE1, ROS, GRP78 along with blocking JAK-STAT route	[129,131]

EDEM-1: ER-associated degradation; ER: Endoplasmic reticulum; ERAD: Endoplasmic reticulum-associated protein degradation; IFN: Interferon; JAK/STAT: Janus kinase (JAK)-signal transducer and activator of transcription (STAT); PI3K/AKT: Phosphatidylinositol 3-kinase (PI3K)/protein kinase B (AKT); NF: No data found; ROS: Reactive oxygen species.

## Future perspective

In conclusion, the increased autophagy activity in the cells infected with coronaviruses is common among all the members of this viral family. The misappropriation of regulatory vesicles in the ERAD process is also common among some members of the coronaviruses. they can also encapsulate the ER membrane to bilayer the vesicles, all of which are responsible for completing their replication process by the mediate-RNA transcription. The process of autophagy and ERAD can be indirectly linked by the LC3 protein bridge, which is due to a decrease or increase in the concentration of LC3-I or LC3-II. Some coronaviruses, such as IBV or PEDV, promote the tendency of the cells to produce autophagosomes by elevating the conversion of LC3-1 to LC3-II. With this evidence, it seems that to inhibit or reduce the proliferation of coronaviruses, it is necessary to understand the exact relationship between the pathogenicity stage and autophagic activity to control the virus replication by affecting this intracellular process autophagic blockers or activators. Thus, according to this evidence, the link between this pathway and coronavirus infection, as well as SARS-CoV-2, can be considered a key point when confronting the current and future coronavirus outbreaks.

The authors’ suggestions are the investigation about the effect of β-coronavirus family infection on increasing the UPR and ERAD pathways and the production of chaperones GRP78, PDI, calmodulin and calreticulin or some proteins such as GADD34 and CHOP.

**Executive summary**

**Endoplasmic reticulum-associated protein degradation & autophagy process machinery**

- The endoplasmic reticulum-associated protein degradation (ERAD) process is a conserved process in the mammalian cells with a role in the disposal of the misfolded protein in the endoplasmic reticulum (ER) lumen.
- The ERAD process is tuned by the disposal of its own regulating factors through the formation of vesicular or autophagosome structure to reach the lysosome stage.
- Autophagy regulates the removal action of misfolded protein and organelle by creating an enclosed bilayer membrane.
- LC3-I is a nonlipid form of LC3, which is found in the cytosol, and LC3-II is the modified arrangement of LC3-I that can bind to the autophagosome membrane covalently, which has a role in ERAD and autophagy activity.

**Coronaviruses can rearrange autophagy & ERAD process**

- SARS-CoV-2 same as other coronaviruses tends to create an enclosed space with a bilayer membrane taken from the ER to replicate its intermediate RNA to direct it toward using the ERAD pathway.
- EDEMosome is considered as an enclosed safe scaffold for their replication of SASR-CoV-2.
- Infected cells have more autophagy activity than normal ones.

**Conclusion**

- Coronaviruses used autophagosome, EDEMosome or another ER membrane formation (double-membrane vesicle, convoluted membrane) as a protective shield to keep itself away from recognition and degradation.
- SARS-CoV-2 can use autophagosomes for its reproduction.
- Coronavirus can provide the opportunity to multiply further through recruitment of the intracellular network such as autophagy and ERAD.
- The increasing activity of autophagy by the coronavirus family and definite assertion about the relationship between SARS-CoV-2 and autophagy need more precise and expanded investigations considering the patients in this regard.
- More comprehensive studies are needed to understand the communication network between autophagy, ERAD and coronaviruses.

**Future perspective**

- Link between this pathway and coronavirus infection as well as SARS-CoV-2 can be considered a key point confronting this and future coronavirus outbreaks.

## References

[1] Lin L-Y, Tran TH. Coronaviruses pandemics: can neutralizing antibodies help? Life Sci. 255, 117836 (2020). 32450171 10.1016/j.lfs.2020.117836 PMC7243778

[2] Escalera-Zamudio M, Gutiérrez B, Thézé J, Pybus OG. A60 revealing the evolution of virulence in RNA viruses. Virus Evol. 5(Suppl. 1), 11–23 (2019).

[3] Soleimanpour S, Yaghoubi A. COVID-19 vaccine: where are we now and where should we go? Expert Rev. Vaccines 20(1), 23–44 (2021). 33435774 10.1080/14760584.2021.1875824 PMC7898300

[4] Yang N, Shen H-M. Targeting the endocytic pathway and autophagy process as a novel therapeutic strategy in COVID-19. Int. J. Biol. Sci. 16(10), 1724 (2020). 32226290 10.7150/ijbs.45498 PMC7098027

[5] Smith M, Wilkinson S. ER homeostasis and autophagy. Essays Biochem. 61(6), 625–635 (2017). 29233873 10.1042/EBC20170092 PMC5869861

[6] Nakka VP, Prakash-babu P, Vemuganti R. Crosstalk between endoplasmic reticulum stress, oxidative stress, and autophagy: potential therapeutic targets for acute CNS injuries. Mol. Neurobiol. 53(1), 532–544 (2016). 25482050 10.1007/s12035-014-9029-6 PMC4461562

[7] Fung TS, Liu DX. Post-translational modifications of coronavirus proteins: roles and function. Future Virol. 13(6), 405–430 (2018). 32201497 10.2217/fvl-2018-0008 PMC7080180

[8] Merulla J, Fasana E, Soldà T, Molinari M. Specificity and regulation of the endoplasmic reticulum-associated degradation machinery. Traffic 14(7), 767–777 (2013). 23521725 10.1111/tra.12068

[9] Olzmann JA, Kopito RR, Christianson JC. The mammalian endoplasmic reticulum-associated degradation system. Cold Spring Harb. Perspect. Biol. 5(9), a013185 (2013). 23232094 10.1101/cshperspect.a013185 PMC3753711

[10] Guerriero CJ, Brodsky JL. The delicate balance between secreted protein folding and endoplasmic reticulum-associated degradation in human physiology. Physiol. Rev. 92(2), 537–576 (2012). 22535891 10.1152/physrev.00027.2011 PMC4162396

[11] Nishikawa S-I, Fewell SW, Kato Y, Brodsky JL, Endo T. Molecular chaperones in the yeast endoplasmic reticulum maintain the solubility of proteins for retrotranslocation and degradation. J. Cell Biol. 153(5), 1061–1070 (2001). 11381090 10.1083/jcb.153.5.1061 PMC2174341

[12] McCracken A, Brodsky J. Recognition and delivery of ERAD substrates to the proteasome and alternative paths for cell survival. Curr. Top. Microbiol. Immunol. 20(1), 17–40 (2006). 10.1007/3-540-28007-3_2 16573235

[13] Auclair SM, Bhanu MK, Kendall DA. Signal peptidase I: cleaving the way to mature proteins. Protein Sci. 21(1), 13–25 (2012). 22031009 10.1002/pro.757 PMC3323777

[14] Ast T, Cohen G, Schuldiner M. A network of cytosolic factors targets SRP-independent proteins to the endoplasmic reticulum. Cell 152(5), 1134–1145 (2013). 23452858 10.1016/j.cell.2013.02.003

[15] Muñiz M, Riezman H. Intracellular transport of GPI-anchored proteins. EMBO 19(1), 10–15 (2000). 10.1093/emboj/19.1.10 PMC1171772 10619839

[16] Christianson JC, Shaler TA, Tyler RE, Kopito RR. OS-9 and GRP94 deliver mutant α1-antitrypsin to the Hrd1–SEL1L ubiquitin ligase complex for ERAD. Nat. Cell Biol. 10(3), 272–282 (2008). 18264092 10.1038/ncb1689 PMC2757077

[17] Gogala M, Becker T, Beatrix B Structures of the Sec61 complex engaged in nascent peptide translocation or membrane insertion. Nature 506(7486), 107–110 (2014). 24499919 10.1038/nature12950

[18] Meusser B, Hirsch C, Jarosch E, Sommer T. ERAD: the long road to destruction. Nat. Cell Biol. 7(8), 766–772 (2005). 16056268 10.1038/ncb0805-766

[19] Bernasconi R, Molinari M. ERAD and ERAD tuning: disposal of cargo and of ERAD regulators from the mammalian ER. Curr. Opin. Cell Biol. 23(2), 176–183 (2011). 21075612 10.1016/j.ceb.2010.10.002 PMC7172097

[20] Le Fourn V, Gaplovska-Kysela K, Guhl B, Santimaria R, Zuber C, Roth J. Basal autophagy is involved in the degradation of the ERAD component EDEM1. Cell. Mol. Life Sci. 66(8), 1434–1445 (2009). 19266160 10.1007/s00018-009-9038-1 PMC11131543

[21] Mueller B, Lilley BN, Ploegh HL. SEL1L, the homologue of yeast Hrd3p, is involved in protein dislocation from the mammalian ER. J. Cell Biol. 175(2), 261–270 (2006). 17043138 10.1083/jcb.200605196 PMC2064567

[22] Bernasconi R, Pertel T, Luban J, Molinari M. A dual task for the Xbp1-responsive OS-9 variants in the mammalian endoplasmic reticulum inhibiting secretion of misfolded protein conformers and enhancing their disposal. J. Biol. Chem. 283(24), 16446–16454 (2008). 18417469 10.1074/jbc.M802272200 PMC3762559

[23] Calì T, Galli C, Olivari S, Molinari M. Segregation and rapid turnover of EDEM1 by an autophagy-like mechanism modulates standard ERAD and folding activities. Biochem. Biophys. Res. Commun. 371(3), 405–410 (2008). 18452703 10.1016/j.bbrc.2008.04.098

[24] Zuber C, Cormier JH, Guhl B, Santimaria R, Hebert DN, Roth J. EDEM1 reveals a quality control vesicular transport pathway out of the endoplasmic reticulum not involving the COPII exit sites. Proc. Natl Acad. Sci. USA 104(11), 4407–4412 (2007). 17360537 10.1073/pnas.0700154104 PMC1810509

[25] Kabeya Y, Mizushima N, Ueno T LC3, a mammalian homologue of yeast Apg8p, is localized in autophagosome membranes after processing. EMBO 19(21), 5720–5728 (2000). 10.1093/emboj/19.21.5720 PMC305793 11060023

[26] Pereira DM, Valentão P, Andrade PB. Tuning protein folding in lysosomal storage diseases: the chemistry behind pharmacological chaperones. J. Biol. Chem. 9(7), 1740–1752 (2018). 10.1039/c7sc04712f PMC5896381 29719681

[27] Ding W-X, Yin X-M. Sorting, recognition and activation of the misfolded protein degradation pathways through macroautophagy and the proteasome. Autophagy 4(2), 141–150 (2008). 17986870 10.4161/auto.5190

[28] Cuervo AM. The plasma membrane brings autophagosomes to life. Nat. Cell Biol. 12(8), 735–737 (2010). 20680002 10.1038/ncb0810-735

[29] Webber JL, Tooze SA. New insights into the function of Atg9. FEBS 584(7), 1319–1326 (2010). 10.1016/j.febslet.2010.01.020 20083107

[30] Orsi A, Razi M, Dooley H Dynamic and transient interactions of Atg9 with autophagosomes, but not membrane integration, are required for autophagy. Mol. Biol. Cell 23(10), 1860–1873 (2012). 22456507 10.1091/mbc.E11-09-0746 PMC3350551

[31] Lane JD, Nakatogawa H. Two ubiquitin-like conjugation systems that mediate membrane formation during autophagy. Essays Biochem. 55, 39–50 (2013). 24070470 10.1042/bse0550039

[32] Sun Q, Fan W, Zhong Q. Regulation of Beclin 1 in autophagy. Autophagy 5(5), 713–716 (2009). 19372752 10.4161/auto.5.5.8524 PMC2789700

[33] Funderburk SF, Wang QJ, Yue Z. The Beclin 1–VPS34 complex–at the crossroads of autophagy and beyond. Trends Cell Biol. 20(6), 355–362 (2010). 20356743 10.1016/j.tcb.2010.03.002 PMC3781210

[34] Yorimitsu T, Klionsky DJ. Autophagy: molecular machinery for self-eating. Cell Death Differ. 12(2), 1542–1552 (2005). 16247502 10.1038/sj.cdd.4401765 PMC1828868

[35] Kang R, Zeh H, Lotze M, Tang D. The Beclin 1 network regulates autophagy and apoptosis. Cell Death Differ. 18(4), 571–580 (2011). 21311563 10.1038/cdd.2010.191 PMC3131912

[36] Fekadu J, Rami A. Beclin-1 deficiency alters autophagosome formation, lysosome biogenesis and enhances neuronal vulnerability of HT22 hippocampal cells. Mol. Neuro. 53(8), 5500–5509 (2016). 10.1007/s12035-015-9453-2 26456737

[37] He C, Levine B. The Beclin 1 interactome. Curr. Opin. Cell Biol. 22(2), 140–149 (2010). 20097051 10.1016/j.ceb.2010.01.001 PMC2854269

[38] Menon MB, Dhamija S. Beclin 1 phosphorylation–at the center of autophagy regulation. Front. Cell Dev. Biol. 6, 137 (2018). 30370269 10.3389/fcell.2018.00137 PMC6194997

[39] Nakatogawa H, Ishii J, Asai E, Ohsumi Y. Atg4 recycles inappropriately lipidated Atg8 to promote autophagosome biogenesis. Autophagy 8(2), 177–186 (2012). 22240591 10.4161/auto.8.2.18373

[40] Maruyama T, Noda NN. Autophagy-regulating protease Atg4: structure, function, regulation and inhibition. J. Antibiot. 71(1), 72–78 (2018). 10.1038/ja.2017.104 PMC5799747 28901328

[41] Thao TTN, Labroussaa F, Ebert N Rapid reconstruction of SARS-CoV-2 using a synthetic genomics platform. Nature 582(7813), 561–565 (2020). 32365353 10.1038/s41586-020-2294-9

[42] Kim D, Lee J-Y, Yang J-S, Kim JW, Kim VN, Chang H. The architecture of SARS-CoV-2 transcriptome. Cell 52(73), 51–65 (2020). 10.1016/j.cell.2020.04.011 PMC7179501 32330414

[43] Maier HJ, Hawes PC, Cottam EM Infectious bronchitis virus generates spherules from zippered endoplasmic reticulum membranes. mBio. 4(5), 21–48 (2013). 10.1128/mBio.00801-13 PMC3812713 24149513

[44] Neuman BW, Angelini MM, Buchmeier MJ. Does form meet function in the coronavirus replicative organelle? Trends Microbiol. 22(11), 642–647 (2014). 25037114 10.1016/j.tim.2014.06.003 PMC7127430

[45] Orenstein JM, Banach B, Baker SC. Morphogenesis of coronavirus HCoV-NL63 in cell culture: a transmission electron microscopic study. Open Infect. Dis. J. 2, 52 (2008). 19844604 10.2174/1874279300802010052 PMC2763395

[46] Ulasli M, Verheije MH, de Haan CA, Reggiori F. Qualitative and quantitative ultrastructural analysis of the membrane rearrangements induced by coronavirus. Cell Microbiol. 12(6), 844–861 (2010). 20088951 10.1111/j.1462-5822.2010.01437.x PMC7159092

[47] Angelini MM, Akhlaghpour M, Neuman BW, Buchmeier MJ. Severe acute respiratory syndrome coronavirus nonstructural proteins 3, 4, and 6 induce double-membrane vesicles. mBio 4(4), 29–57 (2013). 10.1128/mBio.00524-13 PMC3747587 23943763

[48] Lei J, Kusov Y, Hilgenfeld R. Nsp3 of coronaviruses: structures and functions of a large multi-domain protein. Antiviral Res. 149, 58–74 (2018). 29128390 10.1016/j.antiviral.2017.11.001 PMC7113668

[49] Akilesh S, Nicosia RF, Alpers CE Characterizing viral infection by electron microscopy: lessons from the COVID-19 pandemic. Am. J. Pathol. 2(7), 11–20 (2020). 10.1016/j.ajpath.2020.11.003 PMC7678435 33227297

[50] Cottam EM, Maier HJ, Manifava M Coronavirus nsp6 proteins generate autophagosomes from the endoplasmic reticulum via an omegasome intermediate. Autophagy 7(11), 1335–1347 (2011). 21799305 10.4161/auto.7.11.16642 PMC3242798

[51] Benvenuto D, Angeletti S, Giovanetti M Evolutionary analysis of SARS-CoV-2: how mutation of Non-Structural Protein 6 (NSP6) could affect viral autophagy. J. Infect. 12(5), 21–27 (2020). 10.1016/j.jinf.2020.03.058 PMC7195303 32283146

[52] Paul D, Bartenschlager R. Architecture and biogenesis of plus-strand RNA virus replication factories. World J. Virol. 2(2), 32 (2013). 24175228 10.5501/wjv.v2.i2.32 PMC3785047

[53] Wong NA, Saier MH. The sars-coronavirus infection cycle: a survey of viral membrane proteins, their functional interactions and pathogenesis. Int. J. Mol. Sci. 22(3), 1308 (2021). 33525632 10.3390/ijms22031308 PMC7865831

[54] Inoue T, Tsai B. How viruses use the endoplasmic reticulum for entry, replication, and assembly. Cold Spring Harb. Perspect. Biol. 5(1), a013250 (2013). 23284050 10.1101/cshperspect.a013250 PMC3579393

[55] Zhang L, Wang A. Virus-induced ER stress and the unfolded protein response. Front. Plant Sci. 3, 293 (2012). 23293645 10.3389/fpls.2012.00293 PMC3531707

[56] Borczuk AC, Salvatore SP, Seshan SV COVID-19 pulmonary pathology: a multi-institutional autopsy cohort from Italy and New York City. Mod. Pathol. 33(11), 2156–2168 (2020). 32879413 10.1038/s41379-020-00661-1 PMC7463226

[57] Reggiori F, Monastyrska I, Verheije MH Coronaviruses Hijack the LC3-I-positive EDEMosomes, ER-derived vesicles exporting short-lived ERAD regulators, for replication. Cell Host Microbe. 7(6), 500–508 (2010). 20542253 10.1016/j.chom.2010.05.013 PMC7103375

[58] Monastyrska I, Ulasli M, Rottier PJ, Guan J-L, Reggiori F, De Haan CA. An autophagy-independent role for LC3 in equine arteritis virus replication. Autophagy 9(2), 164–174 (2013). 23182945 10.4161/auto.22743 PMC3552881

[59] Bernasconi R, Noack J, Molinari M. Unconventional roles of nonlipidated LC3 in ERAD tuning and coronavirus infection. Autophagy 8(10), 1534–1536 (2012). 22895348 10.4161/auto.21229

[60] Prentice E, Jerome WG, Yoshimori T, Mizushima N, Denison MR. Coronavirus replication complex formation utilizes components of cellular autophagy. J. Biol. Chem. 279(11), 10136–10141 (2004). 14699140 10.1074/jbc.M306124200 PMC7957857

[61] Sharma M, Bhattacharyya S, Nain M Japanese encephalitis virus replication is negatively regulated by autophagy and occurs on LC3-I-and EDEM1-containing membranes. Autophagy 10(9), 1637–1651 (2014). 25046112 10.4161/auto.29455 PMC4206540

[62] Jin R, Zhu W, Cao S Japanese encephalitis virus activates autophagy as a viral immune evasion strategy. PLoS ONE 8(1), e52909 (2013). 23320079 10.1371/journal.pone.0052909 PMC3540057

[63] Giri R, Bhardwaj T, Shegane M Understanding COVID-19 via comparative analysis of dark proteomes of SARS-CoV-2, human SARS and bat SARS-like coronaviruses. Cell. Mol. Life Sci. 9(261), 1–34 (2020). 10.1007/s00018-020-03603-x PMC7382329 32712910

[64] Byun H, Gou Y, Zook A, Lozano MM, Dudley JP. ERAD and how viruses exploit it. Front. Microbiol. 5, 330 (2014). 25071743 10.3389/fmicb.2014.00330 PMC4080680

[65] Frabutt DA, Zheng Y-H. Arms race between enveloped viruses and the host ERAD machinery. Viruses 8(9), 255 (2016). 10.3390/v8090255 PMC5035969 27657106

[66] Jheng J-R, Ho J-Y, Horng J-T. ER stress, autophagy, and RNA viruses. Front. Microbiol. 5, 388 (2014). 25140166 10.3389/fmicb.2014.00388 PMC4122171

[67] Zhao Z, Thackray LB, Miller BC Coronavirus replication does not require the autophagy gene ATG5. Autophagy 3(6), 581–585 (2007). 17700057 10.4161/auto.4782

[68] Prentice E, McAuliffe J, Lu X, Subbarao K, Denison MR. Identification and characterization of severe acute respiratory syndrome coronavirus replicase proteins. J. Virol. 78(18), 9977–9986 (2004). 15331731 10.1128/JVI.78.18.9977-9986.2004 PMC514967

[69] Schneider M, Ackermann K, Stuart M Severe acute respiratory syndrome coronavirus replication is severely impaired by MG132 due to proteasome-independent inhibition of M-calpain. J. Virol. 86(18), 10112–10122 (2012). 22787216 10.1128/JVI.01001-12 PMC3446591

[70] Maier HJ, Britton P. Involvement of autophagy in coronavirus replication. Viruses 4(12), 3440–3451 (2012). 23202545 10.3390/v4123440 PMC3528273

[71] Chen X, Wang K, Xing Y Coronavirus membrane-associated papain-like proteases induce autophagy through interacting with Beclin1 to negatively regulate antiviral innate immunity. Protein Cell 5(12), 912–927 (2014). 25311841 10.1007/s13238-014-0104-6 PMC4259884

[72] Miller K, McGrath ME, Hu Z Coronavirus interactions with the cellular autophagy machinery. Autophagy 5(8), 1–9 (2020). 10.1080/15548627.2020.1817280 PMC7755319 32964796

[73] Cottam EM, Whelband MC, Wileman T. Coronavirus NSP6 restricts autophagosome expansion. Autophagy 10(8), 1426–1441 (2014). 24991833 10.4161/auto.29309 PMC4203519

[74] Guo L, Yu H, Gu W Autophagy negatively regulates transmissible gastroenteritis virus replication. Sci. Rep. 6(1), 1–14 (2016). 27029407 10.1038/srep23864 PMC4814908

[75] Zou D, Xu J, Duan X Porcine epidemic diarrhea virus ORF3 protein causes endoplasmic reticulum stress to facilitate autophagy. Vet. Microbiol. 235, 209–219 (2019). 31383304 10.1016/j.vetmic.2019.07.005 PMC7117398

[76] Gassen NC, Papies J, Bajaj T Analysis of SARS-CoV-2-controlled autophagy reveals spermidine, MK-2206, and niclosamide as putative antiviral therapeutics. bioRxiv (2020) (Epub ahead of print).

[77] Sargazi S, Sheervalilou R, Rokni M, Shirvaliloo M, Shahraki O, Rezaei N. The role of autophagy in controlling SARS-CoV-2 infection: an overview on virophagy-mediated molecular drug targets. Cell Bio. Inter. 5(11), 1–14 (2021). 10.1002/cbin.11609 PMC8251464 33818861

[78] Hemmat N, Asadzadeh Z, Ahangar NK The roles of signaling pathways in SARS-CoV-2 infection; lessons learned from SARS-CoV and MERS-CoV. Arch. Virol. 166(3), 1–22 (2021). 33462671 10.1007/s00705-021-04958-7 PMC7812983

[79] García-Pérez BE, González-Rojas JA, Salazar MI, Torres-Torres C, Castrejón-Jiménez NS. Taming the autophagy as a strategy for treating COVID-19. Cells 9(12), 2679 (2020). 10.3390/cells9122679 PMC7764362 33322168

[80] Yaghoubi A, Amel Jamehdar S, Movaqar A, Milani N, Soleimanpour S. An effective drug against COVID-19: reality or dream? Expert Rev. Respir. Med. 4(15), 1–14 (2020). 10.1080/17476348.2021.1854092 33215942

[81] Pellegrini P, Strambi A, Zipoli C Acidic extracellular pH neutralizes the autophagy-inhibiting activity of chloroquine: implications for cancer therapies. Autophagy 10(4), 562–571 (2014). 24492472 10.4161/auto.27901 PMC3984580

[82] Schrezenmeier E, Dörner T. Mechanisms of action of hydroxychloroquine and chloroquine: implications for rheumatology. Nat. Rev. Rheumatol. 3(16), 1–12 (2020). 10.1038/s41584-020-0372-x 32034323

[83] Solomon VR, Lee H. Chloroquine and its analogs: a new promise of an old drug for effective and safe cancer therapies. Eur. J. Pharmacol. 625(1–3), 220–233 (2009). 19836374 10.1016/j.ejphar.2009.06.063

[84] Cortegiani A, Ippolito M, Ingoglia G, Iozzo P, Giarratano A, Einav S. Update I. A systematic review on the efficacy and safety of chloroquine/hydroxychloroquine for COVID-19. J. Crit. Care 19(2), 29–41 (2020).

[85] Boulware DR, Pullen MF, Bangdiwala AS A randomized trial of hydroxychloroquine as postexposure prophylaxis for Covid-19. N. Engl. J. Med. 11(2), 33–46 (2020). 10.1056/NEJMoa2016638 PMC7289276 32492293

[86] Singh AK, Singh A, Singh R, Misra A. Hydroxychloroquine in patients with COVID-19: a systematic review and meta-analysis. Diab. Vasc. Dis. Res. 19(2), 26–79 (2020).

[87] Gentry CA, Humphrey MB, Thind SK, Hendrickson SC, Kurdgelashvili G, Williams RJ II. Long-term hydroxychloroquine use in patients with rheumatic conditions and development of SARS-CoV-2 infection: a retrospective cohort study. Lancet Rheumatol. 2(11), e689–e697 (2020). 32984847 10.1016/S2665-9913(20)30305-2 PMC7505552

[88] Paton NI, Goodall RL, Dunn DT Effects of hydroxychloroquine on immune activation and disease progression among HIV-infected patients not receiving antiretroviral therapy: a randomized controlled trial. JAMA 308(4), 353–361 (2012). 22820788 10.1001/jama.2012.6936 PMC3821003

[89] Chu C-M, Poon LL, Cheng VC Initial viral load and the outcomes of SARS. CMAJ 171(11), 1349–1352 (2004). 15557587 10.1503/cmaj.1040398 PMC527336

[90] Sher Y, Rabkin B, Maldonado JR, Mohabir P. COVID-19–associated hyperactive intensive care unit delirium with proposed pathophysiology and treatment: a case report. Psychosomatics 61(5), 544 (2020). 32591212 10.1016/j.psym.2020.05.007 PMC7236743

[91] Hanidziar D, Bittner EA. Sedation of mechanically ventilated COVID-19 patients: challenges and special considerations. Anesth. Analg. 12(1), 89–97 (2020). 10.1213/ANE.0000000000004887 PMC7179055 32392023

[92] Troyer EA, Kohn JN, Hong S. Are we facing a crashing wave of neuropsychiatric sequelae of COVID-19? Neuropsychiatric symptoms and potential immunologic mechanisms. Brain Behav. Immun. 22(1), 109–121 (2020). 10.1016/j.bbi.2020.04.027 PMC7152874 32298803

[93] Kraus M, Müller-Ide H, Rückrich T, Bader J, Overkleeft H, Driessen C. Ritonavir, nelfinavir, saquinavir and lopinavir induce proteotoxic stress in acute myeloid leukemia cells and sensitize them for proteasome inhibitor treatment at low micromolar drug concentrations. Leuk. Res. 38(3), 383–392 (2014). 24418752 10.1016/j.leukres.2013.12.017

[94] Shinoda Y, Tagashira H, Bhuiyan MS, Hasegawa H, Kanai H, Fukunaga K. Haloperidol aggravates transverse aortic constriction-induced heart failure via mitochondrial dysfunction. J. Pharmacol. Sci. 131(3), 172–183 (2016). 27435383 10.1016/j.jphs.2016.05.012

[95] Reyskens KM, Essop MF. HIV protease inhibitors and onset of cardiovascular diseases: a central role for oxidative stress and dysregulation of the ubiquitin–proteasome system. Biochim. Biophys. Acta. Mol. Basis Dis. 1842(2), 256–268 (2014). 10.1016/j.bbadis.2013.11.019 24275553

[96] Parker RA, Flint OP, Mulvey R Endoplasmic reticulum stress links dyslipidemia to inhibition of proteasome activity and glucose transport by HIV protease inhibitors. Mol. Pharmacol. 67(6), 1909–1919 (2005). 15755908 10.1124/mol.104.010165

[97] Reyskens KM, Fisher T-L, Schisler JC Cardio-metabolic effects of HIV protease inhibitors (lopinavir/ritonavir). PLoS ONE 8(9), e73347 (2013). 24098634 10.1371/journal.pone.0073347 PMC3787040

[98] Kariya R, Taura M, Suzu S, Kai H, Katano H, Okada S. HIV protease inhibitor lopinavir induces apoptosis of primary effusion lymphoma cells via suppression of NF-κB pathway. Cancer Lett. 342(1), 52–59 (2014). 24012878 10.1016/j.canlet.2013.08.045

[99] Verdugo-Paiva F, Izcovich A, Ragusa M, Rada G, Group C-LOW. Lopinavir/ritonavir for COVID-19: a living systematic review. Medwave 20(6), 84– 99 (2020). 10.5867/medwave.2020.06.7966 32678815

[100] Zhong H, Wang Y, Zhang Z-L Efficacy and safety of current therapeutic options for COVID-19-lessons to be learnt from SARS and MERS epidemic: a systematic review and meta-analysis. Pharmacol. Res. 1(157), 104872 (2020). 10.1016/j.phrs.2020.104872 PMC7192121 32360583

[101] Bhattacharyya A, Kumar S, Sarma P Safety and efficacy of lopinavir/ritonavir combination in COVID-19: a systematic review, meta-analysis, and meta-regression analysis. Pharmacol. Res. 52(4), 313 (2020). 10.4103/ijp.IJP_627_20 PMC7722914 33078733

[102] Zhang JJ, Lee KS, Ang LW, Leo YS, Young BE. Risk factors of severe disease and efficacy of treatment in patients infected with COVID-19: a systematic review, meta-analysis and meta-regression analysis. Clin. Infect. Dis. 5(11), 31–43 (2020). 10.1093/cid/ciaa576 PMC7239203 32407459

[103] Xue E, Zhang Y, Song B, Xiao J, Shi Z. Effect of autophagy induced by dexamethasone on senescence in chondrocytes. Mol. Med. Rep. 14(4), 3037–3044 (2016). 27572674 10.3892/mmr.2016.5662 PMC5042789

[104] Wang XY, Jiao LY, He JL, Fu ZA, Guo RJ. Parathyroid hormone 1–34 inhibits senescence in rat nucleus pulposus cells by activating autophagy via the m-TOR pathway. Mol. Med. Rep. 18(3), 2681–2688 (2018). 29956812 10.3892/mmr.2018.9229 PMC6102631

[105] MENG X-M, HUANG Q-H, HUANG J. Autophagy in peripheral blood CD4+ T lymphocyte of asthma patient autophagy in human peripheral blood T lymphocyte. Chin. J. Lab. Diag. (3), 6 (2007).

[106] Laane E, Tamm KP, Buentke E Cell death induced by dexamethasone in lymphoid leukemia is mediated through initiation of autophagy. Cell Death Differ. 16(7), 1018–1029 (2009). 19390558 10.1038/cdd.2009.46

[107] Zhang S, Liu Y, Liang Q. Low-dose dexamethasone affects osteoblast viability by inducing autophagy via intracellular ROS. Mol. Med. Rep. 17(3), 4307–4316 (2018). 29363725 10.3892/mmr.2018.8461 PMC5802204

[108] Liu W, Zhao Z, Na Y, Meng C, Wang J, Bai R. Dexamethasone-induced production of reactive oxygen species promotes apoptosis via endoplasmic reticulum stress and autophagy in MC3T3-E1 cells. Int. J. Mol. Med. 41(4), 2028–2036 (2018). 29393368 10.3892/ijmm.2018.3412 PMC5810234

[109] Troncoso R, Paredes F, Parra V Dexamethasone-induced autophagy mediates muscle atrophy through mitochondrial clearance. Cell Cycle. 13(14), 2281–2295 (2014). 24897381 10.4161/cc.29272 PMC4111682

[110] Das I, Png CW, Oancea I Glucocorticoids alleviate intestinal ER stress by enhancing protein folding and degradation of misfolded proteins. J. Exp. Med. 210(6), 1201–1216 (2013). 23650437 10.1084/jem.20121268 PMC3674691

[111] Horby P, Lim WS, Emberson JR Dexamethasone in hospitalized patients with Covid-19-preliminary report. NEJM 7(13), 47–56 (2020).

[112] Johnson RM, Vinetz JM. Dexamethasone in the management of covid-19. BMJ 1(4), 47–66 (2020). 10.1136/bmj.m2648 32620554

[113] Schmeisser H, Bekisz J, Zoon KC. New function of type I IFN: induction of autophagy. J. Interferon Cytokine Res. 34(2), 71–78 (2014). 24428799 10.1089/jir.2013.0128 PMC3924851

[114] Zhang X, Zeng Y, Qu Q PD-L1 induced by IFN-γ from tumor-associated macrophages via the JAK/STAT3 and PI3K/AKT signaling pathways promoted progression of lung cancer. J. Clin. Oncol. 22(6), 1026–1033 (2017). 10.1007/s10147-017-1161-7 28748356

[115] Maher SG, Sheikh F, Scarzello AJ IFN-α and IFN-λ differ in their antiproliferative effects and duration of JAK/STAT signaling activity. Cancer Biol. Ther. 7(7), 1109–1115 (2008). 18698163 10.4161/cbt.7.7.6192 PMC2435218

[116] Zhao J, Wang M-L, Li Z Interferon-alpha-2b induces autophagy in hepatocellular carcinoma cells through Beclin1 pathway. Cancer Biol. Ther. 11(1), 64 (2014). 10.7497/j.issn.2095-3941.2014.01.006 PMC3969804 24738040

[117] Law AH-Y, Lee DC-W, Yuen K-Y, Peiris M, Lau AS-Y. Cellular response to influenza virus infection: a potential role for autophagy in CXCL10 and interferon-alpha induction. Cell. Mol. Immunol. 7(4), 263–270 (2010). 20473322 10.1038/cmi.2010.25 PMC4003230

[118] Lee HK, Lund JM, Ramanathan B, Mizushima N, Iwasaki A. Autophagy-dependent viral recognition by plasmacytoid dendritic cells. Science 315(5817), 1398–1401 (2007). 17272685 10.1126/science.1136880

[119] Münz C. Enhancing immunity through autophagy. Annu. Rev. Immunol. 27, 423–449 (2009). 19105657 10.1146/annurev.immunol.021908.132537

[120] Li Y, Zhu H, Zeng X Suppression of autophagy enhanced growth inhibition and apoptosis of interferon-β in human glioma cells. Mol. Neur. 47(3), 1000–1010 (2013). 10.1007/s12035-013-8403-0 23329343

[121] Li P, Du Q, Cao Z Interferon-gamma induces autophagy with growth inhibition and cell death in human hepatocellular carcinoma (HCC) cells through interferon-regulatory factor-1 (IRF-1). Cancer Lett. 314(2), 213–222 (2012). 22056812 10.1016/j.canlet.2011.09.031 PMC3487386

[122] Barrera M-J, Aguilera S, Castro I Pro-inflammatory cytokines enhance ERAD and ATF6α pathway activity in salivary glands of Sjögren’s syndrome patients. J. Autoimmun. 75, 68–81 (2016). 27461470 10.1016/j.jaut.2016.07.006

[123] Pirot P, Eizirik DL, Cardozo AK. Interferon-γ potentiates endoplasmic reticulum stress-induced death by reducing pancreatic beta cell defence mechanisms. Diabetologia 49(6), 1229 (2006). 16604358 10.1007/s00125-006-0214-7

[124] Lokugamage KG, Hage A, Schindewolf C, Rajsbaum R, Menachery VD. SARS-CoV-2 is sensitive to type I interferon pretreatment. BioRxiv 94(23), (2020). 10.1128/JVI.01410-20 PMC7654262 32938761

[125] Channappanavar R, Fehr AR, Vijay R Dysregulated type I interferon and inflammatory monocyte-macrophage responses cause lethal pneumonia in SARS-CoV-infected mice. Cell Host Microbe. 19(2), 181–193 (2016). 26867177 10.1016/j.chom.2016.01.007 PMC4752723

[126] Zhang Q, Bastard P, Liu Z Inborn errors of type I IFN immunity in patients with life-threatening COVID-19. Science 370(6515), 102–124 (2020). 10.1126/science.abd4570 PMC7857407 32972995

[127] Zhou Q, Chen V, Shannon CP Interferon-α2b treatment for COVID-19. Front. Immunol. 11, 1061 (2020). 32574262 10.3389/fimmu.2020.01061 PMC7242746

[128] Hu Z-J, Xu J, Yin J-M Lower circulating interferon-gamma is a risk factor for lung fibrosis in COVID-19 patients. Front. Immunol. 11, 2348 (2020). 10.3389/fimmu.2020.585647 PMC7550399 33133104

[129] Łuczkowska K, Rogińska D, Ulańczyk Z, Paczkowska E, Schmidt CA, Machaliński B. Molecular mechanisms of bortezomib action: novel evidence for the miRNA–mRNA interaction involvement. Int. J. Mol. Sci. 21(1), 350 (2020). 10.3390/ijms21010350 PMC6981510 31948068

[130] Richardson PG, Anderson KC. Bortezomib: a novel therapy approved for multiple myeloma. Neurology 1(10), 596–600 (2003). 16258456

[131] Longhitano L, Tibullo D, Giallongo C Proteasome inhibitors as a possible therapy for SARS-CoV-2. Int. J. Mol. Sci. 21(10), 3622 (2020). 10.3390/ijms21103622 PMC7279248 32443911

[132] Angelini MM, Neuman BW, Buchmeier MJ. Untangling membrane rearrangement in the nidovirales. DNA Cell Biol. 33(3), 122–127 (2014). 24410069 10.1089/dna.2013.2304 PMC3942677

[133] Gassen NC, Niemeyer D, Muth D SKP2 attenuates autophagy through Beclin1-ubiquitination and its inhibition reduces MERS-Coronavirus infection. Nature 10(1), 1–16 (2019). 10.1038/s41467-019-13659-4 PMC6920372 31852899

[134] Randhawa PK, Scanlon K, Rappaport J, Gupta MK. Modulation of autophagy by SARS-CoV-2: a potential threat for cardiovascular system. Front. Physiol. 11, 1560 (2020). 10.3389/fphys.2020.611275 PMC7734100 33329064

[135] Yaghoubi A, Amel Jamehdar S, Movaqar A, Milani N, Soleimanpour S. An effective drug against COVID-19: reality or dream? Expert Rev. Respir. Med. 1, 15–60 (2020). 10.1080/17476348.2021.1854092 33215942

[136] Subramani S, Malhotra V. Non-autophagic roles of autophagy-related proteins. EMBO 14(2), 143–151 (2013). 10.1038/embor.2012.220 PMC3566844 23337627

[137] Gözüaçık D. Novel ATG5 interactors in the control of basic autophagy and mitophagy. The Scientific and Technological Research Council of Turkey 23(1), 41–52 (2016).

[138] Choi Y, Bowman JW, Jung JU. Autophagy during viral infection—a double-edged sword. Nat. Rev. Microbiol. 16(6), 341–354 (2018). 29556036 10.1038/s41579-018-0003-6 PMC6907743

[139] Gratton R, Tricarico PM, Guimaraes RL, Celsi F, Crovella S. Lopinavir/ritonavir treatment induces oxidative stress and caspase-independent apoptosis in human glioblastoma U-87 MG cell line. Curr. HIV Res. 16(2), 106–112 (2018). 29804534 10.2174/1570162X16666180528100922

